# Optical visualization of ultrathin mica flakes on semitransparent gold substrates

**DOI:** 10.1186/1556-276X-8-305

**Published:** 2013-07-02

**Authors:** Aurora Dols-Perez, Xavier Sisquella, Laura Fumagalli, Gabriel Gomila

**Affiliations:** 1 Nanobioelec Group, Institute for BioEngineering of Catalonia (IBEC), c/Baldiri i Reixac 15-21, Barcelona 08028, Spain; 2 Departament d’Electrònica, Universitat de Barcelona, c/Martí i Franquès 1, Barcelona 08028, Spain; 3 Nanotechnology Platform, Barcelona Science Park, c/Josep Samitier 1-5, Barcelona 08028, Spain; 4 Current address: The Walter and Eliza Hall Institute of Medical Research, Parkville, Melbourne 3052, Australia

**Keywords:** Ultrathin layers, Optical microscopy, Conductive AFM, Metallic substrate

## Abstract

We show that optical visualization of ultrathin mica flakes on metallic substrates is viable using semitransparent gold as substrates. This enables to easily localize mica flakes and rapidly estimate their thickness directly on gold substrates by conventional optical reflection microscopy. We experimentally demonstrate it by comparing optical images with atomic force microscopy images of mica flakes on semitransparent gold. Present results open the possibility for simple and rapid characterization of thin mica flakes as well as other thin sheets directly on metallic substrates.

## Background

Thin and ultrathin mica flakes have been recently proposed as a promising dielectric material for graphene- and carbon nanotube-based electronics [1-3]. Among the outstanding properties of thin mica sheets, one finds high dielectric constant, atomically flat surface, chemical and mechanical stability, the possibility to obtain single atomic sheets [2], and excellent adhesion to graphene with no ripples [4]. For some applications such as the use of mica sheets as gate dielectric, mica flakes are directly in contact with a metallic surface [3]. It is known that the properties of some ultrathin sheet materials like graphene can be greatly affected by its contact with a metallic material, and therefore it is fundamental to understand whether this is also the case for ultrathin mica flakes. To develop such investigations, it would be advantageous to have a simple optical technique capable to localize mica flakes directly on metallic surfaces and determine their thickness *in situ* similarly as it can be done on Si0_2_/Si substrates [2,3]. However, the possibility to optically detect mica flakes on metallic substrates has not been reported yet.

In this paper, we precisely address this issue and demonstrate that thin mica flakes can be visualized on semitransparent gold substrates, and their thickness can be estimated by optical microscopy. We show that the optical contrast is largely enhanced using *semitransparent* metallic substrates, instead of opaque metallic substrates, which enable accurate characterization of ultrathin mica flakes.

### Theoretical background

**Figure 1:**
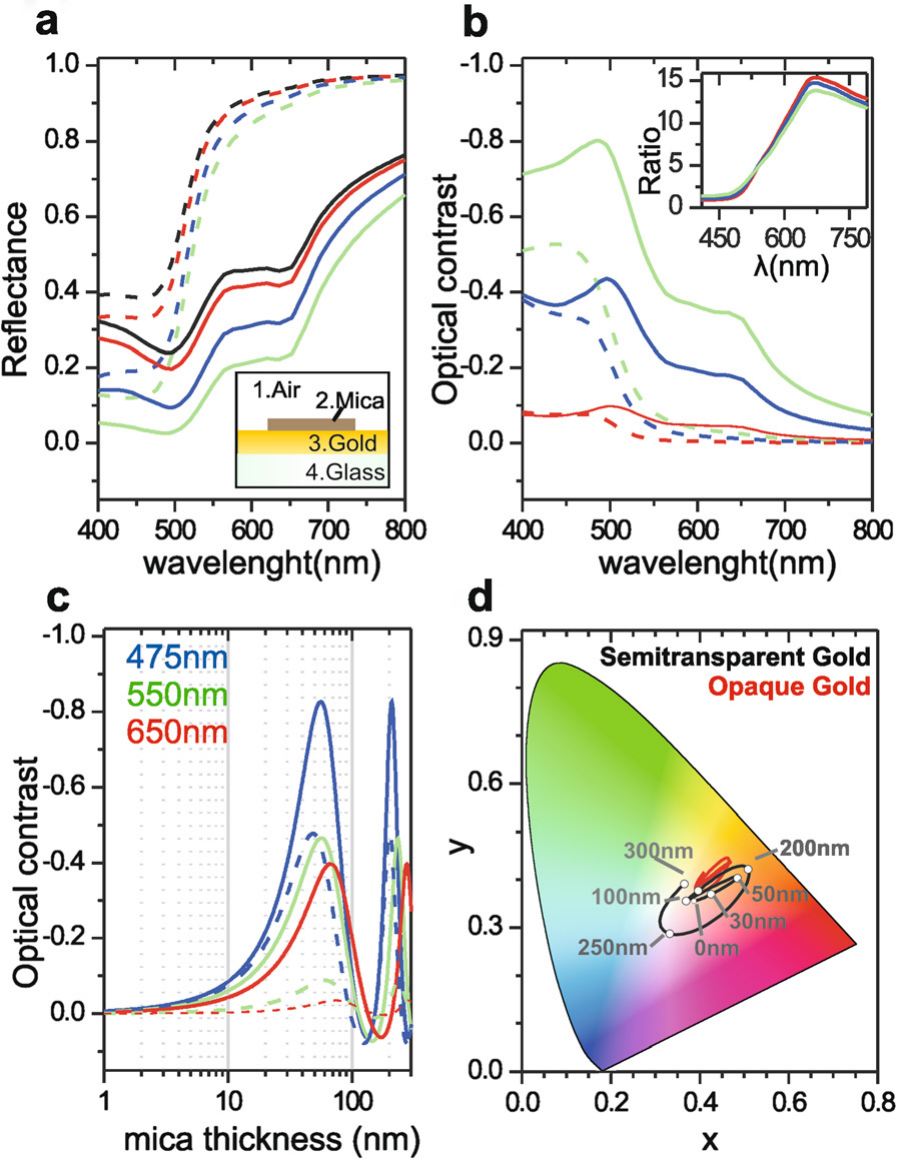
**Calculated reflectance spectra, optical contrasts, and color evolution of the mica flakes. (a)** Calculated reflectance spectra of mica (colored lines) and gold (black lines) in the structure shown in the inset as a function of the wavelength of visible light. Mica thicknesses are 0 nm (black lines, bare gold), 10 nm (red lines), 30 nm (blue lines), and 50 nm (green lines). Gold layer thicknesses are 20 nm (continuous lines) and 300 nm (dashed lines). Inset: schematic representation of the layered structure analyzed. **(b)** Optical contrast in reflection mode between the mica and gold parts of the sample as a function of the wavelength of light for the same cases considered in (a). Inset: ratio between the contrasts for the two gold layer thicknesses considered. **(c)** Optical contrast in reflection mode as a function of mica thickness for three representative wave lengths, 475 nm (blue lines), 550 nm (green lines), and 650 nm (red lines), and two gold layer thickness, 20 nm (continuous lines) and 300 nm (dashed lines). **(d)** Evolution of the mica color (lines) as a function of its thickness in the *xy* chromatographic space for the case of semitransparent (black line) and opaque (red line) gold substrates.

We consider the mica-gold system schematically shown in the inset of Figure 1a. It consists of a thin mica flake on a metallic layer supported by a glass slab. According to the transfer matrix formalism [5], the reflectance for normal incidence of the mica and gold in the considered structure can be calculated as:

(1) $$R_{\mathrm{mica}}\left(\lambda ,d_{2},d_{3}\right)=\frac{{\left|T_{1,0}^{14}\right|}^{2}}{{\left|T_{0,0}^{14}\right|}^{2}}\;R_{\mathrm{gold}}\left(\lambda ,d_{2},d_{3}\right)=\frac{{\left|{\bar{T}}_{1,0}^{14}\right|}^{2}}{{\left|{\bar{T}}_{0,0}^{14}\right|}^{2}},$$

where

(2) $$T^{14}=T^{12}\times T^{2}\times T^{23}\times T^{3}\times T^{34}{\;\bar{T}}^{14}=T^{13}\times T^{3}\times T^{34},$$

with

(3) $$T^{j}=\left(\begin{array}{cc} e^{i\frac{2\pi }{\lambda }{\tilde{n}}_{j}d_{j}} & 0\\ 0 & e^{-i\frac{2\pi }{\lambda }{\tilde{n}}_{j}d_{j}} \end{array}\right)\;T^{\mathrm{jk}}=\frac{1}{t_{\mathrm{jk}}}\times \left(\begin{array}{cc} 1 & r_{\mathrm{jk}}\\ r_{\mathrm{jk}} & 1 \end{array}\right)$$

and

(4) $$r_{\mathrm{jk}}=\frac{{\tilde{n}}_{k}-{\tilde{n}}_{j}}{{\tilde{n}}_{k}+{\tilde{n}}_{j}};\;t_{\mathrm{jk}}=\frac{2{\tilde{n}}_{j}}{{\tilde{n}}_{k}+{\tilde{n}}_{j}}.$$

Here, *λ* is the wavelength of light, and *d*_2_ and *d*_3_ are the thicknesses of the mica and gold layers, respectively. For simplicity, the glass substrate is assumed to be infinitely thick. Moreover, *ñ*_*j*_ = *n*_*j*_ − *ik*_*j*_ is the complex index of refraction of material *j* (where we use *j* = 1 for air, *j* = 2 for mica, *j* = 3 for gold, and *j* = 4 for glass) with *n* being the real part (index of refraction) and *k* the imaginary part (extinction coefficient). We have taken *ñ*_1_ = 1 + *i*0 for air, *ñ*_2_ = 1.55 + *i*0 for mica [2], *ñ*_3_(*λ*) = *n*(*λ*) − *ik*(*λ*) for gold with tabulated values taken from [6], and *ñ*_4_ = 1.52 + *i*0 for glass. From the reflectance, we can define the optical contrast as:

(5) $$C_{R}\left(\lambda ,d_{2},d_{3}\right)=\frac{R_{\mathrm{mica}}-R_{\mathrm{gold}}}{R_{\mathrm{mica}}+R_{\mathrm{gold}}}.$$

In Equations 1 to 5, we have considered a non-null transmission of the gold layer in order to include the case of semitransparent gold.

Figure 1a shows the reflectance spectra for the gold substrate and the mica flakes obtained from Equations 1 to 4. We have considered two representative thicknesses for the gold layer, that is, 20 nm (continuous lines) and 300 nm (dashed lines), and different mica thicknesses, namely 0 nm (black lines, bare gold), 10 nm (red lines), 30 nm (blue lines), and 50 nm (green lines). The gold thickness of 20 nm represents a semitransparent layer, enabling some light transmission, while the 300-nm-thick gold represents an opaque layer (no light transmission). By comparing the black lines (gold substrate) with the colored lines (mica flakes of different thickness), we observe that the presence of thin mica flakes can significantly modify the reflectivity of the gold substrates and that the reflectance varies as a function of the mica thickness. This means that the presence of mica sheets, and their thickness, should be measurable by reflection optical microscopy directly on gold substrates. The precision of the thickness measurement depends on the thickness of the gold layer and on the wavelength range. Indeed, as shown in Figure 1b, where the optical contrast is given as a function of the wavelength, the optical contrast for opaque gold substrates (dashed lines) is only appreciable in the short wavelength range (*λ* < 550 nm), while, in the case of semitransparent gold substrates (continuous lines), it is significant practically in the full range of visible light and again higher in the blue-green region of the spectrum. Importantly, the optical contrast on semitransparent gold is enhanced by a factor between 5 and 16 with respect to the case of an opaque gold substrate for wavelengths *λ* > 550 nm (see the inset of Figure 1b where the ratio between the contrasts is given). These results indicate that enhanced visualization and thickness estimation of mica flakes can be achieved on semitransparent gold substrates.

The dependence of the optical contrast on the thickness of the mica flakes is shown in Figure 1c for three representative wavelengths (*λ* = 475, 550, and 650 nm) and for the two thickness values of the gold layer, i.e., 20 nm (continuous lines, semitransparent gold) and 300 nm (dashed lines, opaque gold). The optical contrast shows an oscillatory behavior characteristic of multilayered structures [5], with an enhanced signal for semitransparent gold (compare continuous and dashed lines of the same color). The oscillatory behavior of the optical contrast is due to an oscillatory behavior of the mica reflectance spectrum, which can be translated into an oscillatory change in the color of the mica flakes perceived by the human eye. Indeed, for a standard observer the chromaticity of the color of a material under white illumination can be defined by the parameters *x* and *y* given by [7]:

(6) $$x=\frac{X}{X+Y+Z};\;y=\frac{Y}{X+Y+Z},$$

where the tristimulus *X*, *Y*, and *Z* are defined from the reflectance spectrum as:

(7) $$X=\int_{380}^{780}\bar{x}\left(\lambda \right)\times R\left(\lambda \right)\;;\;Y=\int_{380}^{780}\bar{y}\left(\lambda \right)\times R\left(\lambda \right)\;;\;Z=\int_{380}^{780}\bar{z}\left(\lambda \right)\times R\left(\lambda \right)\;.$$

Here, $\bar{x}$, $\bar{y}$, and $\bar{z}$ are the so-called color matching functions of a standard observer [7]. In Figure 1d, we show the calculated evolution of the chromaticity of the mica flakes' color in the *xy* chromatographic space as a function of the mica thickness in the 0- to 300-nm range. The black and red lines correspond to the semitransparent and opaque gold layers, respectively. According to these results, we expect a gradual change of color as the mica thickness increases in the thin range below approximately 50 nm. This gradual change is almost reversed back for thicker layers, between 50 and 100 nm, and then evolves to larger and fastest chromaticity changes with the thickness from 100 to 300 nm. In the case of an opaque gold substrate (red line in Figure 1d), the evolution of the chromaticity of the mica flakes is qualitatively similar but restricted to a narrower space of colors, thus making increasingly difficult to achieve a precise optical characterization on this type of substrates. It is worth mentioning that the theoretical contrast that can be achieved on semitransparent gold substrates is between half and three halves of the contrast that can be achieved on SiO_2_ substrates [2,3], in which single mica layers can be detected. This makes reasonable the detection of a few mica layer sheets on semitransparent gold substrates.

## Methods

We verified the theoretical predictions discussed above by fabricating thin mica flakes on semitransparent gold films and characterizing them by optical and atomic force microscopy. To obtain mica flakes on semitransparent gold layers, we adapted the procedure previously developed for the fabrication of flat gold substrates [8]. First, an approximately 20-nm-thick layer of gold was deposited on a thick and freshly cleaved mica substrate using a vacuum system UNIVEX 450 (Salem, NH, USA) at 4 × 10^−4^ mbar by thermal evaporation, and then a glass support (Menzel-Gläser, Braunschweig, Germany; 0.8-mm thick, 8 × 8 mm^2^ area, and index of refraction *n* = 1.517) has been glued with an epoxy resin (EPO-TEK H74-110, index of refraction before curing *n* = 1.569, Epoxy Technology Inc., Billerica, MA, USA) on the gold-evaporated mica. Finally, the glass support has been detached from the mica substrate, exposing the gold surface in contact with the mica. In this process of mechanically removing the mica, some mica flakes of various thicknesses and widths remained attached to the gold surface. This preparation method, with respect to other preparation in which mica flakes are transferred to the substrates, has the main advantage of ensuring a very clean and atomically flat interface between the mica flake and the gold substrate. The gold layer surface in contact with the epoxy layer shows a root mean square roughness of approximately 2.5 nm as measured by atomic force microscopy. Compared to the theoretical structure used in the calculations (inset of Figure 1a), the experimental structure displays an additional layer between the gold and the glass, i.e., the epoxy resin. Since the index of refraction of the resin is very close to that of the glass substrate, its explicit effect can be neglected in the calculations.

The gold surfaces with thin mica flakes on it were then characterized by optical reflection microscopy using an AxioImager A1m (Zeiss, Oberkochen, Germany) mounted with an AxioCam ERc5s camera. Moreover, conductive atomic force microscopy (C-AFM) images were taken with a commercial AFM (Nanotec Electronica, S.L., Madrid, Spain) with a custom-made current amplifier [9]. C-AFM measurements simultaneously provide conductivity and topography of the mica flakes. This enabled us first to distinguish mica flakes from gold by measuring the insulating behavior of the mica as opposed to conductive gold and then to precisely measure the thickness of the flakes from topography. We used doped diamond AFM tips (CDT-FMR, Nanosensors, Neuchatel, Switzerland; spring constant of 2.1 N/m). All C-AFM measurements were done in contact mode with 100 mV applied at room temperature with approximately 0% relative humidity controlled by dry N_2_(g) flow. A resistance of approximately 100 MΩ was connected in series with the substrate to limit the current. Image processing was performed with WSxM software (Nanotec Electronica) [10].

## Results and discussion

**Figure 2:**
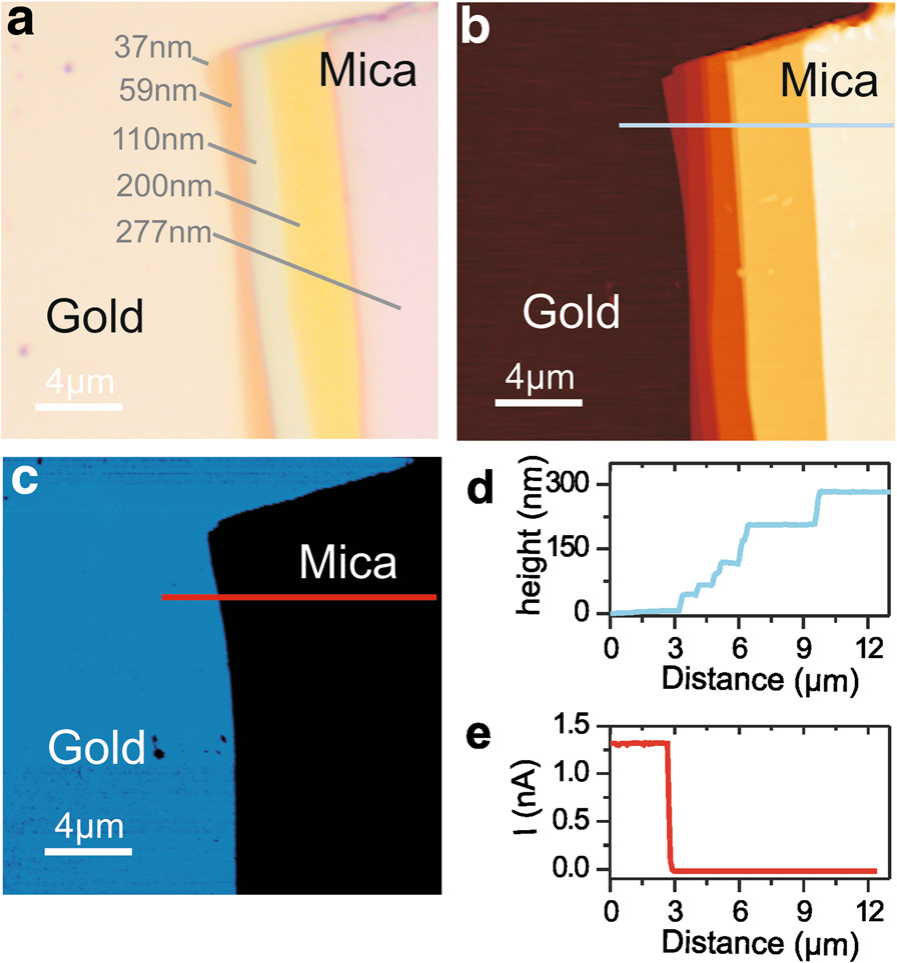
**Reflection optical microscopy, AFM topography, and conduction images of mica flakes on semitransparent gold. (a)** Reflection optical microscopy image of a staircase mica flake with thicknesses in the 37- to 277-nm range on a semitransparent gold layer. **(b)** AFM topography and **(c)** conduction images of the same area. **(d)** Topographic and **(e)** current profiles along the lines indicated in **(b)** and **(c)**, respectively.

Figure 2 shows the optical and C-AFM images of a staircase mica flake with thickness in the 37- to 277-nm range on a semitransparent gold substrate. The optical reflection image (Figure 2a) clearly shows the presence of the mica flake that displays distinct colors depending on the thickness, as obtained by AFM imaging and profiling (Figure 2b,d). The C-AFM image (Figure 2c) and current profile (Figure 2e) clearly confirm the conductive and insulating behavior of the gold and mica regions, respectively. These results demonstrate that mica flakes can be visualized by optical microscopy directly on gold substrates with a remarkable optical contrast and remarkable dependence of the mica color on the mica thickness. In particular, in the range of thicknesses reported in Figure 1, the mica exhibits a relatively large color space with increasing sensitivity to the thickness in the 100- to 300-nm range. Furthermore, we note that the specific colors shown by the different mica thicknesses are in quasi-quantitative agreement with the colorimetric results shown in Figure 1d.

**Figure 3:**
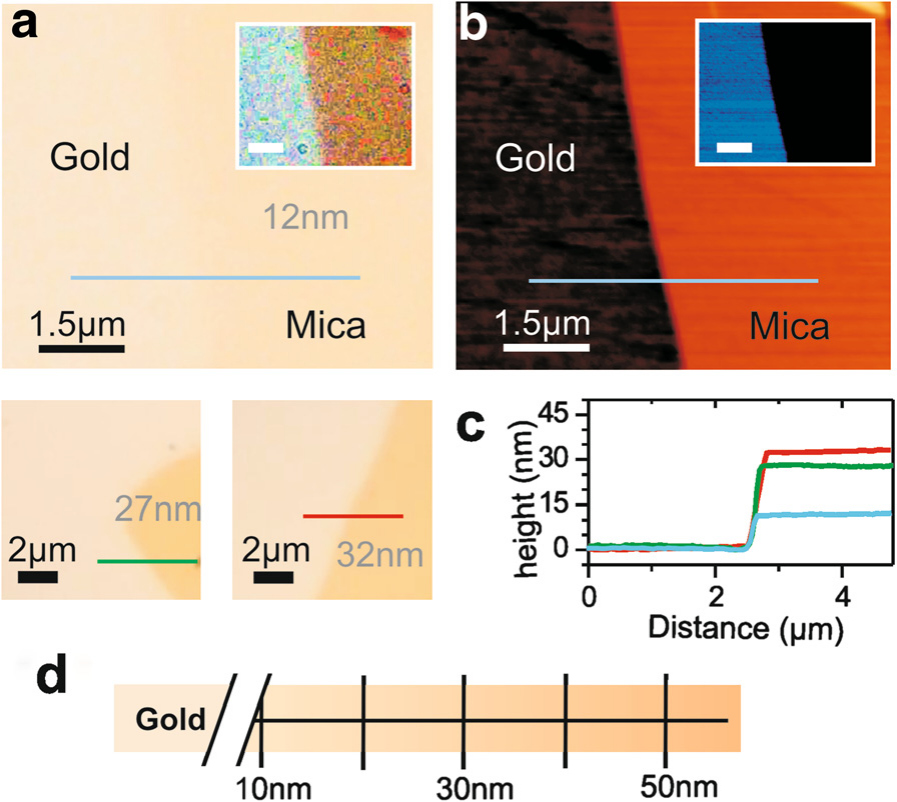
**Reflection optical microscopy, AFM topography, conduction images, and approximate color scale of ultrathin mica sheets on gold. (a)** Reflection optical microscopy images of three mica sheets on semitransparent gold substrates with thicknesses in the 12- to 32-nm range. Inset: same as the main image but with artificially enhanced contrast. **(b)** AFM topographic image of the approximately 12-nm mica flake. Inset: corresponding conductive image. **(c)** Topographic profiles of the mica flakes shown in **(a)** taken along the indicated lines. **(d)** Approximate color scale for mica sheets as a function of the thickness with thickness in the 10- to 50-nm range.

Figure 3a shows the optical images of three mica flakes of smaller thicknesses (12- to 32-nm range). As before, the thickness and the insulating nature of the mica flakes were measured by C-AFM. An example of topographic and conduction images for the 12-nm-thick flake is shown in Figure 3b, while the topographic profiles of the three flakes are given in Figure 3c. The contrast achieved on the 12-nm-thin mica flakes is high enough to reasonably expect the detection of thinner mica flakes if present on the sample (note that direct observation from the eyepieces of the optical microscope provides a better contrast as compared to the camera-recorded image. An artificially enhanced contrast image is shown in the inset of Figure 3a in order to show that mica flakes are easily identifiable). Results demonstrate that mica flakes down to a few layers' thickness can be detected on a semitransparent gold substrate by optical microscopy in agreement with the theoretical calculations in Figure 1c. Furthermore, the evolution of the mica color as a function of the mica thickness in this range of thicknesses (Figure 3d) is gradual and with chromatic values in quasi-quantitative agreement with the theoretical predictions in Figure 1d, thus still allowing reasonable thickness estimation.

## Conclusions

In summary, we have shown that thin mica sheets can be optically visualized on gold substrates and that the optical contrast can be greatly enhanced using semitransparent gold layers as compared to using opaque gold substrates. We found that for thick sheets (thickness > 100 nm), the optical color shows a remarkable dependence on the sheet thickness, thus enabling to easily estimate it by optical microscopy. For thinner mica flakes (thickness < 50 nm) the mica colors change more gradually, but it remains possible to estimate the mica thickness with reasonable accuracy down to a few mica layers. These results should allow building a color chart for mica thicknesses on semitransparent gold layers similar to the one derived for Si0_2_ on Si [7] or for other ultrathin sheets such as graphene, graphite, or other materials [11-13] on Si0_2_/Si. The proposed technique will greatly facilitate the investigations of the properties of ultrathin mica flakes in direct contact with metallic materials, which until now have not been explored. Additionally, the present results also open the possibility to enable the visualization of other thin sheets, like graphene, directly on metallic supports [14].

## Competing interests

The authors declare that they have no competing interests.

## Authors’ contributions

ADP analyzed the samples by AFM and optical microscopy and suggested the study. XS produced the samples. GG developed the theoretical calculations. LF and GG coordinated the investigation. ADP and GG jointly wrote the manuscript. All authors read and approved the final version of the manuscript.
